# Supplementary material for: Experimental Autoimmune Encephalomyelitis of Mice: IgGs from the Sera of Mice Hydrolyze miRNAs
Source: Int J Mol Sci. 2023 Feb 23;24(5):4433. doi: 10.3390/ijms24054433 (PMC10002156; doi:10.3390/ijms24054433)
Supplement: Supplementary file 1 [file ijms-24-04433-s001.zip › ijms-2180975-supplementary.pdf]

Supplementary methods

Immunization of mice (Part I)

Immunization of C57BL/6 mice with MOG was carried out as described in previously published protocols [1-3]. On day 1 (zero time), mice were immunized by injection of 10 μg of MOG per mouse in the back, two times in the left and right side using 20 μl of Freund's complete adjuvant containing Pertussis toxin (400 ng/mouse; *Mycobacterium tuberculosis*). The next day an additional 20 μl of Pertussis Toxin (400 ng / mouse) was injected in a similar way.

IgG purification (Part II).

Electrophoretically and immunologically homogeneous mouse IgGs were obtained by sequential chromatography of the serum proteins on Protein G-Sepharose and following fast protein liquid chromatography (FPLC) gel filtration as described previously [1-3]. The serum protein (0.4–0.6 ml) was loaded onto a 1-ml protein G-Sepharose column equilibrated in buffer A (150 mM NaCl, 50 mM Tris-HCl, pH 7.5). The column was washed by buffer A to zero optical density (A_{280}). Proteins adsorbed non-specifically were eluted with the same buffer (15 ml) but containing 1% Triton X-100 and 0.3M NaCl and the column was washed with buffer A to zero optical density. The total IgGs fraction was eluted with 0.1 M glycine-HCl (pH 2.6), the column fractions were collected to cooled tubes containing 50 ml of 0.5M Tris-HCl (pH 9.0), and finally each fraction was additionally neutralized with this buffer, concentrated for additional purification.

The purified IgG was incubated in acidic glycine-HCl buffer (pH 2.6) to disrupt non-covalent interactions and subjected to FPLC gel filtration on a Superdex 200 HR 10/30 column (Pfizer, New York,NY) using the BioCA workstation (Applied Biosystems, Foster City, CA) [1-3]. Abs were incubated for 20 min at 25° C in 0.1 M buffer (pH 2.6) containing 0.3 M NaCl and then subjected to the gel filtration on the column equilibrated in buffer A. The fractions of separated IgGs were collected and dialyzed against 20mM Tris-HCl (pH 7.5) containing 50 mM NaCl.

In order to protect Abs preparations from bacterial and viral contamination, they were filtered through Millex syringe-driven filter units (0.2 μm) and kept in sterilized tubes. Incubation of standard bacterial medium with stored Abs preparations did not lead to the formation of colonies.

Supplementary Figures

All Figures was taken and combined from the following articles:

1. Doronin, V. B., Parkhomenko, T.A., Korablev, A., Toporkova, L.B., Lopatnikova, J.A., et al. “Changes different parameters, lymphocyte proliferation and hematopoietic progenitor colony formation in EAE mice treated with myelin oligodendrocyte glycoprotein.” Journal of Cellular and Molecular Medicine, vol. 20, no. 1, pp. 81–94, 2016.

2. Aulova, K.S., Toporkova, L.B., Lopatnikova, J.A., Alshevskaya, A A., Sennikov, S.V., et al. “Changes in haematopoietic progenitor colony differentiation and proliferation and the production of different abzymes in EAE mice treated with DNA.”Journal of Cellular and Molecular Medicine, vol. 21, no. 12, pp. 3795–3809, 2017.

3. Aulova, K.S., Toporkova, L.B., Lopatnikova, J.A., Alshevskaya, A.A., Sedykh, S.E., et al. Changes in cell differentiation and proliferation lead to production of abzymes in EAE mice treated with DNA-Histone complexes. “Journal of Cellular and Molecular Medicine” vol. 22, pp. 5816-5832, 2018.

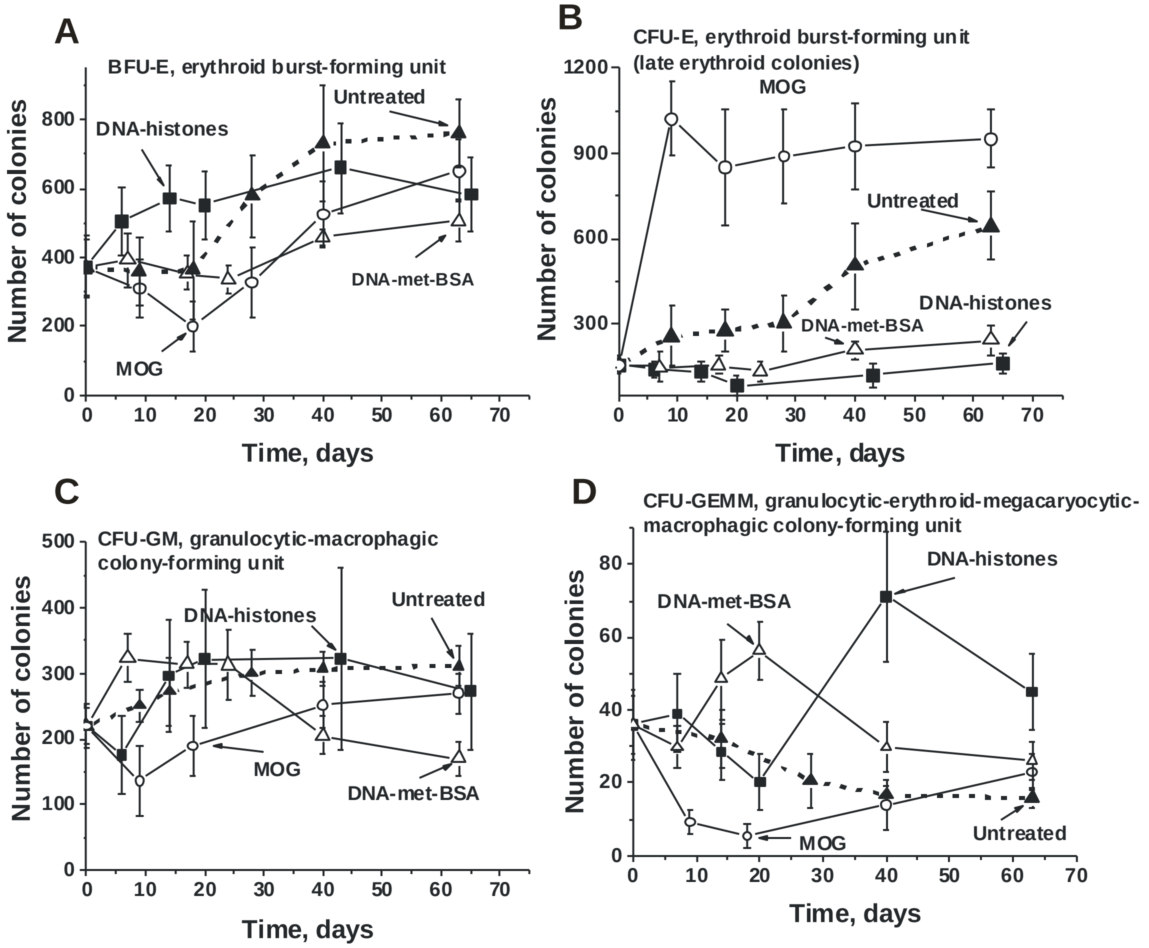

Supplementary Figure S1. Changes over time in a number of mice brain BFU-E (**A**), CFU-E (**B**), CFU-GM (**C**), and CFU-GEMM (**D**) forming colony units are shown for untreated mice, as well as after their treatment with DNA-histone, a complex of DNA

with methylated BSA (DNA-met-BSA), and MOG [1-3]. Immunogens used are shown on Panels **A-D**.

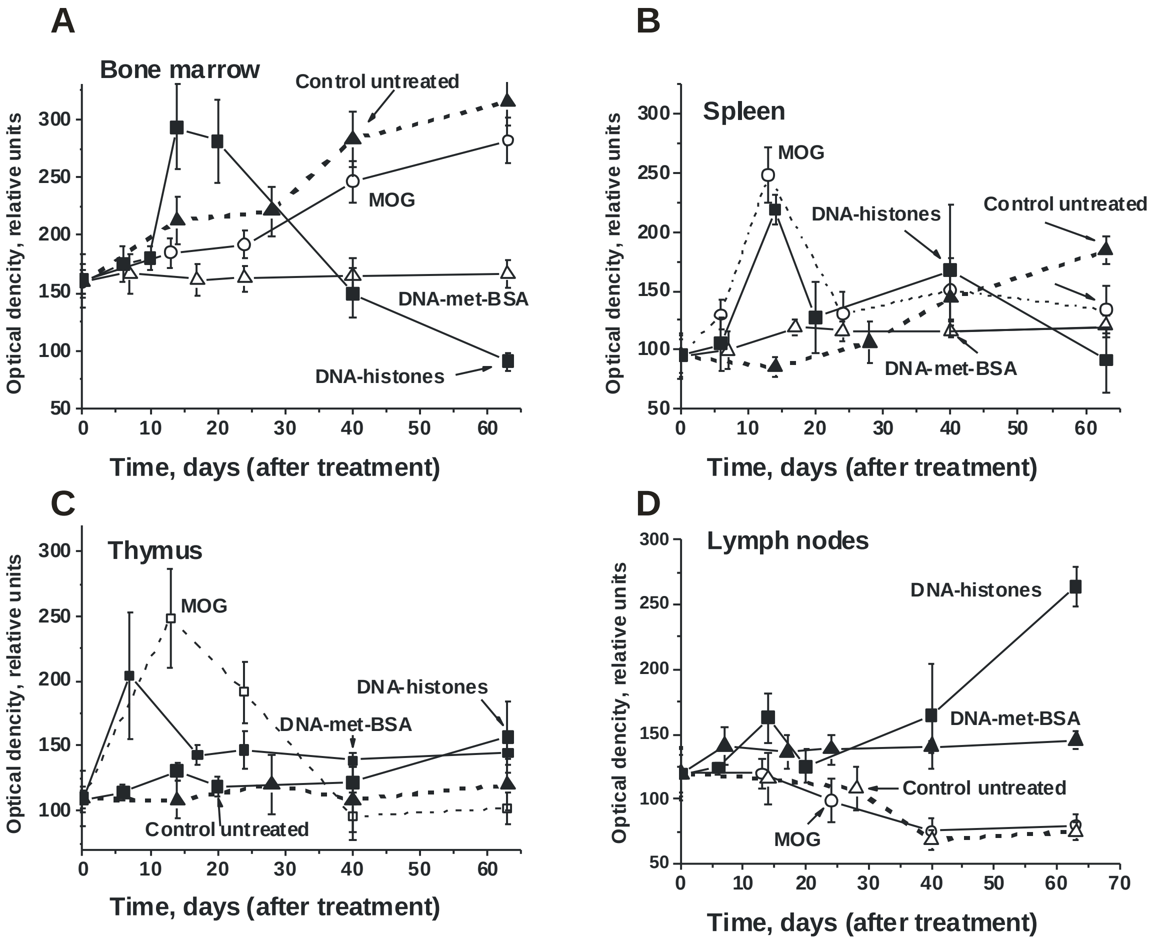

Supplementary Figure S2. The average over time changes in the optical density reflecting the relative amount of lymphocytes in bone marrow (**A**), spleen (**B**), thymus (**C**), and lymph nodes (**D**) are shown for untreated mice, as well as after their treatment with DNA-histone, a complex of DNA with methylated BSA (DNA-met-BSA), and MOG. Immunogens used are shown on Panels **A-D**. The error in the optical density estimation for each mouse for all groups (with seven mice per group) from three independent experiments did not exceed 7–10% [1-3].

An analysis in time of relative activity of IgGs during spontaneous and MOG-induced development of EAE in C57BL/6 was previously carried out [1-3]. It was shown that immunization of mice with MOG leads to a change in the relative activity of antibodies in the hydrolysis of DNA, MBP, and MOG. The Figures in the changes in the activities in C57BL/6 mice are given below

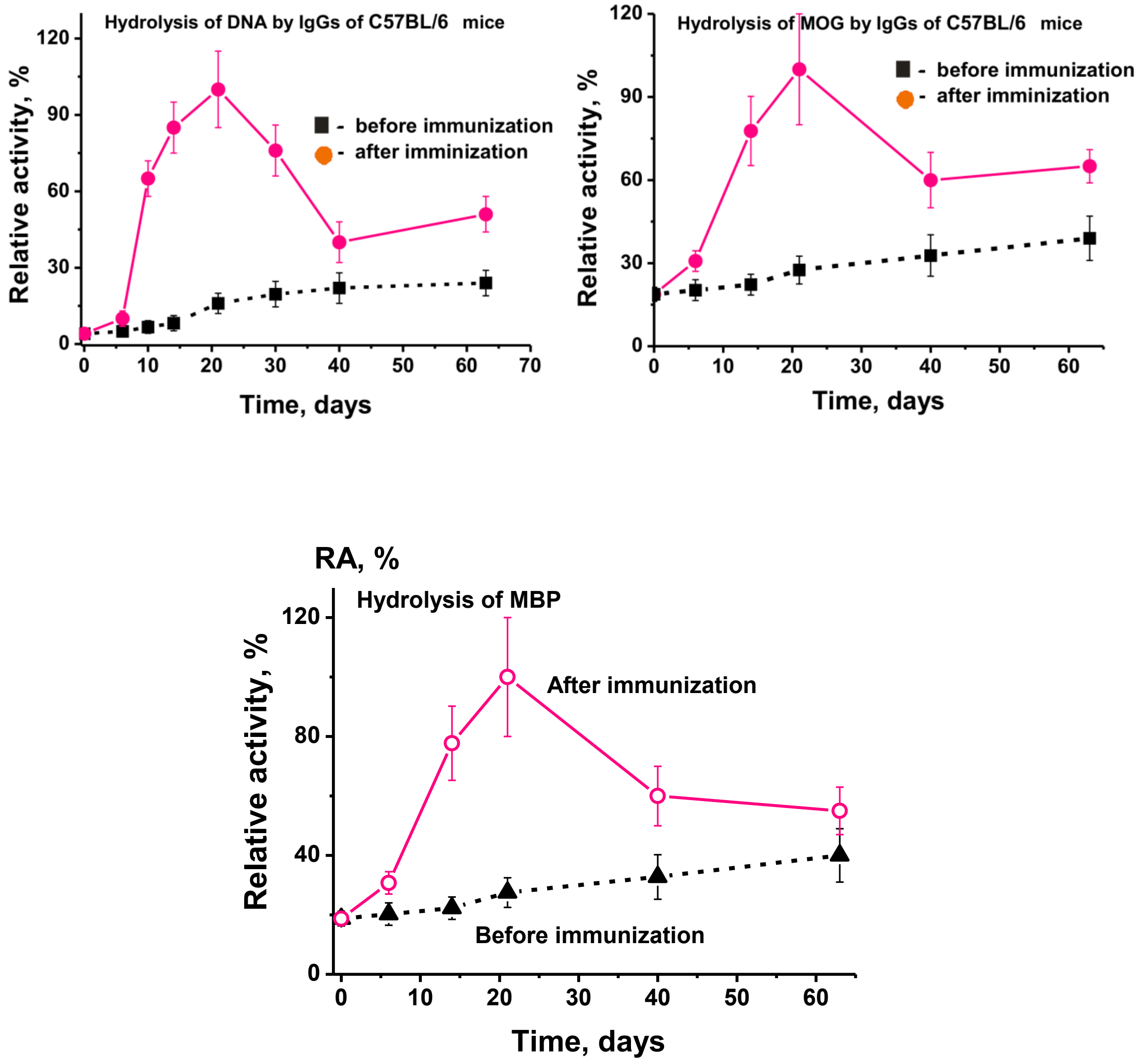

Supplementary Figure S3

Overtime changes of the average relative activities of IgGs of untreated and MOG-treated C57BL/6 mice (7 mice of each group) [1,2].
